# Pharmacokinetics and Acute Safety Evaluation of Tryptanthrin, A Bioactive Constituent of Indigo naturalis, Following Intravenous Administration in Mice

**DOI:** 10.1002/jat.70169

**Published:** 2026-04-08

**Authors:** Hyung Jin Kim, Gabsik Yang, Jun Ho Lee

**Affiliations:** ^1^ Department of Natural Products & Biotechnology Jeonbuk Science College Jeongeup Jeollabuk‐do Republic of Korea; ^2^ Lee Gil Ya Cancer and Diabetes Institute Gachon University Incheon Republic of Korea; ^3^ College of Korean Medicine Gachon University Seongnam Republic of Korea; ^4^ Department of Pharmaceutical & Engineering, College of BIT Convergence Seowon University Cheongju‐si Republic of Korea

**Keywords:** acute toxicity, *Indigo naturalis*, intravenous administration, natural products, pharmacokinetics, tryptanthrin

## Abstract

The use of *Indigo naturalis* in treating inflammatory diseases is clinically restricted by severe adverse events, including pulmonary arterial hypertension and hepatotoxicity. Tryptanthrin, a major indoloquinazoline alkaloid of *Indigo naturalis*, exhibits potent anti‐inflammatory activity; however, its specific safety profile relative to the crude extract remains unclear. This study aimed to elucidate the intravenous pharmacokinetics and acute safety of purified tryptanthrin to validate its potential as a safer pharmacological alternative. Following a single intravenous injection of 30 mg/kg—a dose range relevant to its pharmacological efficacy—in ICR mice, plasma concentrations were quantified using a validated LC–MS/MS method. Tryptanthrin exhibited rapid systemic distribution and biphasic elimination, characterized by a large volume of distribution (V_ss_ 23.1 L/kg) and rapid clearance (6.54 L/h/kg), indicating extensive tissue penetration and efficient systemic elimination. Importantly, over a 14‐day observation period, no mortality, behavioral abnormalities, or significant alterations in hepatic (AST, ALT, total bilirubin) and renal (BUN, creatinine) biochemical parameters were observed. These findings indicate that purified tryptanthrin does not induce the acute hepatic or renal toxicity frequently associated with crude *Indigo naturalis* preparations. This study provides the first pharmacokinetic characterization and safety validation of tryptanthrin at a therapeutic dose, supporting its preclinical development as a safe, single‐chemical entity for systemic administration.

## Abstract 2

This study evaluates the intravenous pharmacokinetics and acute safety of tryptanthrin, an anti‐inflammatory alkaloid from *Indigo naturalis*, to address the severe toxicity associated with its crude extract. Following a 30 mg/kg intravenous dose in mice, tryptanthrin demonstrated rapid systemic clearance and extensive distribution without inducing acute hepatotoxicity or nephrotoxicity. These findings indicate that isolating tryptanthrin successfully dissociates pharmacological efficacy from the crude matrix's adverse effects, presenting it as a significantly safer therapeutic alternative for inflammatory diseases.

## 1 Introduction

Tryptanthrin is an indoloquinazoline alkaloid originally isolated from *Indigo naturalis* (Qingdai), a traditional medicinal material widely used in East Asia. This compound has demonstrated diverse biological properties, including antimicrobial (Duca et al. 2019; Yang et al. 2024), anti‐inflammatory (He et al. 2024; Pergola et al. 2012; Micallef et al. 2002; Kim et al. 2024), and anticancer activities (Liao et al. 2013; Zhou 2024; Chang et al. 2015). *Indigo naturalis* has shown significant therapeutic efficacy in treating inflammatory diseases such as ulcerative colitis and psoriasis in multiple randomized controlled trials (Lin et al. 2007; Lin et al. 2008; Lin et al. 2012; Cheng et al. 2017; Lin et al. 2018; Naganuma et al. 2018; Lin et al. 2020; Uchiyama et al. 2020; Naganuma et al. 2020).

However, the clinical application of crude *Indigo naturalis* is severely restricted by safety concerns, particularly adverse events such as hepatic dysfunction and pulmonary arterial hypertension (PAH) (Nishio et al. 2016; Nishio et al. 2018; Naganuma et al. 2019; Misumi et al. 2019; Hiraide et al. 2021; Masaki et al. 2021). These toxic effects are believed to originate from other chemical constituents, such as indigo and indirubin, rather than from tryptanthrin itself. Therefore, validating tryptanthrin as a safe, purified alternative is a critical toxicological priority to overcome the clinical limitations of the crude extract.

Although tryptanthrin has been widely studied for its pharmacological potential, its systemic safety profile remains uncharacterized. Previous studies have mainly focused on oral administration of *Indigo naturalis* extracts (Li et al. 2012; Zhang et al. 2017), and no detailed PK or safety evaluation of isolated tryptanthrin has been reported. Establishing the intravenous pharmacokinetic properties is an essential prerequisite for its translational development, as understanding the IV pharmacokinetics is fundamental for elucidating absorption, distribution, metabolism, and excretion (ADME) characteristics and for defining safe and effective dosage regimens (Jeong et al. 2020; Parker and Davey 1992; Singh 2006). While oral administration is common, intravenous delivery is crucial for acute inflammatory conditions requiring rapid onset, yet no IV PK or safety data currently exist. Evaluation of intravenous pharmacokinetics is critical to determine the fundamental disposition characteristics of the drug without the confounding variables of gastrointestinal absorption and first‐pass metabolism. Therefore, this route provides the necessary baseline for a comprehensive safety and pharmacokinetic evaluation.

Accordingly, this study was designed to characterize the intravenous pharmacokinetic profile of tryptanthrin and to evaluate its acute safety in ICR mice. By defining the elimination kinetics and confirming the absence of acute toxicity at a therapeutically relevant dose, these findings provide fundamental data for risk assessment and support the development of tryptanthrin as a pharmacologically active natural compound with an improved safety profile compared with its source material.

## 2 Materials and Methods

### 2.1 Chemicals and Reagents

Tryptanthrin (CAS No.: 13220‐57‐0, purity 99.89%, molecular weight 248.24) was purchased from Sigma‐Aldrich (St. Louis, MO, United States). The compound identity was confirmed through spectroscopic analysis and comparison with authentic standards. Warfarin (purity ≥ 98%, code no. A2250, lot no. SLCN1941) was supplied by Woojung Bio Inc. (Suwon, Republic of Korea) and used as an internal standard for bioanalytical method validation. HPLC‐grade acetonitrile, methanol, and water were purchased from Burdick & Jackson. Dimethyl sulfoxide (DMSO, 99.97% purity) and formic acid (99.5% purity) were obtained from Sigma‐Aldrich and Fisher Chemical, respectively. All other chemicals and reagents were of analytical grade.

### 2.2 Experimental Animals and Ethical Approval

Male ICR (CrlOri: CD1) mice (7 weeks old, 25–30 g) were supplied by a licensed vendor (Koatech Inc., Eumseong, Republic of Korea). Animals were maintained under controlled environmental conditions (22°C ± 2°C; 55% ± 5% humidity; 12 h light/dark cycle) with free access to standard laboratory chow and water. After 1 week of acclimatization, mice were randomly assigned to experimental groups. All experimental procedures were conducted in accordance with the guidelines for animal experimentation established by the institutional ethics committee and were approved by the Institutional Animal Care and Use Committee (IACUC) of Woojung Bio Inc. (Suwon, Republic of Korea; approval no. IACUC2303‐042). The study protocol complied with international guidelines for animal welfare and the principles of replacement, reduction, and refinement (3Rs). A total of 55 mice were used in this study. The animals were randomly assigned to two main experimental sets: the pharmacokinetic study group and the acute safety assessment group.

### 2.3 Intravenous Administration and Sample Collection

Tryptanthrin was dissolved in a vehicle consisting of a 1:1 (v/v) mixture of methanol and DMSO to prepare a stock solution, which was then diluted with water to achieve a final concentration of 3 mg/mL with 10% DMSO content. To exclude any potential influence of the vehicle on the safety outcomes, a vehicle‐treated control group administered the same solvent mixture was included in the acute toxicity assessment. The compound was administered intravenously at a dose of 30 mg/kg via the lateral tail vein.

The dose of 30 mg/kg was selected to represent a therapeutically relevant level for initial safety and pharmacokinetic evaluation, consistent with intravenous doses employed in preclinical investigations of similar bioactive natural compounds (Mohammed et al. 2025; Di Cesare Mannelli et al. 2018; Arunachalam et al. 2024). This dosage allows for the assessment of systemic safety margins within a range likely to exert pharmacological activity, prioritizing the validation of safety at effective concentrations over lethal dose determination in this first systematic intravenous study.

Animals were fasted for 15 h prior to dosing with free access to water. Blood samples (approximately 400 μL each) were collected via cardiac puncture under light isoflurane anesthesia at predetermined time points: 0.083, 0.25, 0.5, 1, 2, 4, 8, 12, and 24 h post‐administration (*n* = 5 per time point). This sampling duration of 24 h was considered sufficient as it covers approximately six times the terminal half‐life of tryptanthrin (approx. 4 h), ensuring adequate characterization of the terminal elimination phase. Cardiac puncture was selected to ensure the collection of sufficient blood volume for accurate bioanalysis. Since this terminal procedure necessitated a sparse sampling design, pharmacokinetic parameters were calculated based on the mean plasma concentration‐time profile to minimize the impact of inter‐individual variability. The collected blood was immediately transferred to K2‐EDTA (ethylenediaminetetraacetic acid) tubes to prevent coagulation. Blood samples were then centrifuged at 1000 g for 10 min at 4°C, and plasma was separated and stored at −80°C until analysis.

### 2.4 Quantification of Tryptanthrin in Plasma

Plasma concentrations of tryptanthrin were determined using a validated LC–MS/MS method developed specifically for this ethnopharmacological investigation. The LC–MS/MS system consisted of an AB Sciex LC‐40UHPLC system coupled to an AB Sciex QTRAP 5500 + triple quadrupole mass spectrometer with Analyst Ver 1.7.2 data system (AB Sciex, Framingham, MA, United States). For sample preparation, plasma samples (5 μL) were mixed with 95 μL of methanol containing internal standard (warfarin, 50 ng/mL) for protein precipitation. After vortex mixing for 60 s at 2500 rpm and centrifugation at 15,520 g for 10 min at 4°C, 2 μL of the supernatant was injected into the LC–MS/MS system.

Chromatographic separation was achieved using a Phenomenex Kinetex C18 column (2.1 × 50 mm, 2.6 μm) maintained at 50°C. The mobile phase consisted of 0.1% formic acid in water (A) and acetonitrile (B) with gradient elution: initial 30% B, held for 0.5 min, increased to 70% B over 0.5 min, held at 70% B until 1.5 min, then returned to 30% B at 2.0 min and maintained until 3.0 min. The flow rate was 0.5 mL/min with an injection volume of 2 μL. Mass spectrometric detection was performed in positive electrospray ionization (ESI+) mode using multiple reaction monitoring (MRM). The MRM transitions were *m/z* 249.0 → 130.1 for tryptanthrin and *m/z* 308.9 → 163.1 for warfarin (internal standard). MS parameters included the following: source temperature 750°C, ion spray voltage 4000 V, with optimized declustering potential, entrance potential, collision energy, and collision cell exit potential for each compound.

The analytical method was validated according to regulatory guidelines, demonstrating linearity over the concentration range of 10–10,000 ng/mL with a lower limit of quantification (LLOQ) of 10 ng/mL and correlation coefficient (r^2^) > 0.995. The established LLOQ of 10 ng/mL provided sufficient sensitivity to characterize the terminal elimination phase, as the mean plasma concentration at the final sampling point (24 h) was 17.5 ng/mL, remaining quantifiably above the lower limit. Signal‐to‐noise ratio at LLOQ was 14.8. Back‐calculated concentrations showed accuracy ranging from 88.8% to 113.7% across all calibration levels, and inter‐ and intra‐day precision was within 15% at all quality control levels.

### 2.5 Pharmacokinetic Analysis

Non‐compartmental pharmacokinetic analysis was performed using WinNonlin software (Certara, Princeton, NJ, United States). This model‐independent method was selected to determine pharmacokinetic parameters based directly on the observed data without making assumptions about a specific compartmental structure. The area under the plasma concentration–time curve from time zero to the last quantifiable concentration (AUC_0−last_) and to infinity (AUC_0−_∞) was calculated using the linear trapezoidal rule. The terminal elimination half‐life (t1/2) was determined by log‐linear regression of the terminal phase. Total body clearance (CL) was calculated as Dose/AUC_0−_∞, and the steady‐state volume of distribution (V_ss_) was determined using the relationship V_ss_ = CL × MRT, where MRT represents the mean residence time (MRT).

### 2.6 Acute Toxicity and Clinical Observations

For the acute safety assessment, a separate set of 10 mice was used. The animals were randomly divided into two groups (*n* = 5 per group): the vehicle control group and the tryptanthrin‐treated group. For acute safety assessment, additional groups of mice were monitored for 14 days following intravenous administration of tryptanthrin at the same dose used for pharmacokinetic studies. Clinical signs, mortality, and body weight changes were recorded daily throughout the observation period. On Day 14, blood samples were collected under anesthesia for comprehensive serum biochemistry analysis. Parameters evaluated included markers of hepatic function (aspartate aminotransferase [AST], alanine aminotransferase [ALT], alkaline phosphatase, total bilirubin), renal function (blood urea nitrogen [BUN], creatinine), and general metabolic status (total protein, albumin, glucose, total cholesterol, triglycerides). Biochemical measurements were performed using an automated chemistry analyzer (Hitachi 7180, Tokyo, Japan) with commercial assay kits (Asan Pharm, Seoul, Republic of Korea) according to the manufacturer's instructions. The criteria for toxicity evaluation included mortality, clinical signs (behavioral changes), body weight fluctuations, and significant alterations in plasma biochemical parameters compared with the vehicle control group.

### 2.7 Statistical Analysis

The sample size of five animals per group (*n* = 5) was determined to provide adequate statistical power to detect significant differences at an α level of 0.05, consistent with standard sample sizes employed in similar pharmacokinetic investigations. Data are presented as mean ± standard deviation (SD). Statistical analyses were performed using SPSS 18.0 software (SPSS Inc., Chicago, IL, United States). For safety parameters, differences between treatment and control groups were assessed using unpaired Student's *t*‐test or one‐way analysis of variance (ANOVA) followed by Dunnett's multiple comparison test, as appropriate. A *p*‐value < 0.05 was considered statistically significant.

## 3 Results

### 3.1 Plasma Concentration‐Time Profile

**FIGURE 1:**
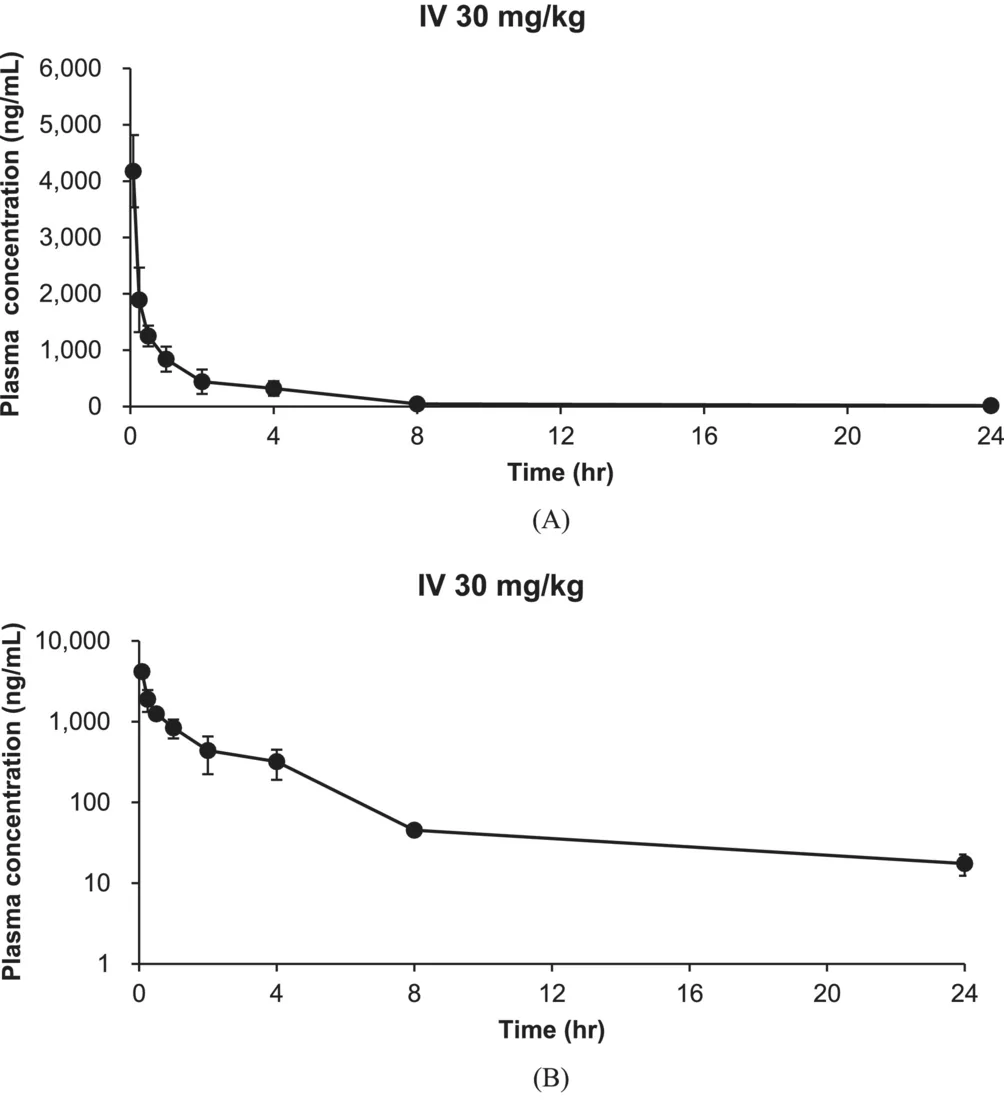
Plasma concentration‐time profile of tryptanthrin following a single intravenous administration of 30 mg/kg in ICR mice. Data are presented as mean ± SD (*n* = 5). Inset shows the semi‐logarithmic plot.

After a single intravenous administration of tryptanthrin at the therapeutic dose of 30 mg/kg, plasma concentrations declined in a biphasic manner, showing rapid distribution followed by elimination (Figure 1). The mean maximum plasma concentration (C_max_) was 4175.7 ± 641.1 ng/mL at 0.083 h, confirming immediate systemic exposure. Detectable concentrations were maintained up to 24 h, approaching the lower limit of quantification at the final time point.

### 3.2 Method Validation and Analytical Performance

The LC–MS/MS assay exhibited acceptable accuracy and precision for quantitative determination of tryptanthrin in mouse plasma. Calibration curves were linear between 10 and 10,000 ng/mL (*r* ^2^ > 0.995), and accuracy ranged from 88.8% to 113.7% across calibration levels. The intra‐ and inter‐day precision was within 15%, confirming suitability for pharmacokinetic analysis (Table 1).

**TABLE 1:** Back‐calculated concentrations and accuracy of calibration standards for tryptanthrin in mouse plasma.

#	Back‐calculated concentration of calibration standards (ng/mL)							
	10	50	100	200	500	1000	5000	10,000
1	9.6	54.8	113.7	218.9	471.3	926.9	4751.9	8877.9
Mean	9.6	54.8	113.7	218.9	471.3	926.9	4751.9	8877.9
SD	—	—	—	—	—	—	—	—
CV (%)	—	—	—	—	—	—	—	—
Accuracy (%)	**96.4**	**109.7**	**113.7**	**109.5**	**94.3**	**92.7**	**95.0**	**88.8**

#	Calibration curve parameters		
	Slope	Intercept	Coefficient of correlation
1	**0.00021**	**0.00017**	**0.99500**
Mean	0.00021	0.00017	0.99500
SD	—	—	—
CV (%)	—	—	—

*Note:* This table displays the back‐calculated concentrations of calibration standards used to quantify tryptanthrin plasma levels, ranging from 10 to 10,000 ng/mL. The table shows accuracy values between 88.8% and 113.7%, indicating the reliability and precision of the calibration process used in the pharmacokinetic analysis. Bold entries indicate the quantifier ions.

Abbreviations: CV, coefficient of variation; SD, standard deviation.

### 3.3 Plasma Concentration Data and Variability

Mean plasma concentrations of tryptanthrin at each sampling point are presented in Table 2. Concentrations decreased from 4175.7 ng/mL at 0.083 h to 17.5 ng/mL at 24 h. The coefficient of variation among individuals remained below 20% for most time points, indicating moderate inter‐animal variability typical for intravenous pharmacokinetic studies.

**TABLE 2:** Plasma concentrations of tryptanthrin at various time points following intravenous administration of 30 mg/kg in ICR mice.

No.	IV_Time (h)	Concentration (unit: ng/mL, *n* = 5)	
		IV 30 mg/kg	
		Mean	SD
1	0.083	4175.7	641.1
2	0.25	1893.0	572.9
3	0.5	1250.9	184.6
4	1	840.3	220.9
5	2	439.8	216.1
6	4	319.7	129.6
7	8	45.2	5.4
8	24	17.5	5.1

*Note:* The table highlights the rapid rise in concentration, peaking at 4175.7 ng/mL at 0.083 h, followed by a steep decline to 17.5 ng/mL at 24 h. These values reflect the fast distribution and clearance of tryptanthrin from the bloodstream.

Abbreviation: IV, intravenous.

### 3.4 Pharmacokinetic Parameters

Non‐compartmental analysis produced an AUC_0_–last of 4483.9 ng·h/mL and AUC_0_–∞ of 4586.2 ng·h/mL. The MRT was 3.5 h, with total body clearance of 109 mL/min/kg, reflecting rapid systemic elimination, and a steady‐state volume of distribution (V_ss_) of 23.1 L/kg, indicative of extensive tissue distribution (Table 3).

**TABLE 3:** Pharmacokinetic parameters of tryptanthrin after intravenous administration in ICR mice.

Parameter	G1_IV 30 mg/kg
AUC_(last)__ng/mL·h	4483.9 ± 669.30
AUC_(inf)__ng/mL·h	4586.2 ± 685.00
MRT_(inf)__h	3.5 ± 0.38
C_max__ng/mL	4175.7 ± 641.10
T_max__obs_h	0.083 ± 0.0000
CL_(obs)__ mL/min/kg	109.0 ± 16.50
V_ss(obs)__L/kg_IV	23.1 ± 3.30
V_z(obs)__L/kg_PO	38.3 ± 14.30
t_1/2__h	4.057 ± 1.060

*Note:* Data are presented as mean ± SD (*n* = 5).

Abbreviations: AUC_(inf)_, AUC with extrapolation to infinity; AUC_(last)_, AUC from time zero to the last time point; CL, clearance; C_max_, maximal plasma concentration; MRT, mean retention time; T_max__obs, observed time to maximum concentration; t_1/2_, terminal half‐life; V_ss_, Volume of distribution at steady state; V_z_, Volume of distribution.

### 3.5 Acute Safety Assessment

Consistent with the favorable safety profile anticipated for the purified compound, no mortality or behavioral signs of toxicity were observed in any mice during the 14‐day post‐treatment period. Body weight changes remained within physiological ranges, showing no significant difference compared with the vehicle control group.

Importantly, serum biochemistry analysis on Day 14 confirmed the absence of hepatic and renal injury. Hepatic function markers (AST, ALT, total bilirubin), which are typically elevated in *Indigo naturalis*‐induced liver injury, remained comparable with those of the normal control group. Similarly, renal function indices (BUN, creatinine) and metabolic parameters (total protein, albumin, glucose, lipids) showed no significant alterations (Table 4). These results demonstrate that intravenous tryptanthrin, at the therapeutically relevant dose, does not induce acute systemic toxicity.

**TABLE 4:** Biochemical analysis of plasma from ICR mice following intravenous injection of tryptanthrin.

	Vehicle control group	Tryptanthrin‐injected group
AST (IU/L)	24.3 ± 1.76	25.1 ± 1.44
ALT (IU/L)	24.1 ± 1.94	27.4 ± 3.97
BUN (mg/dL)	9.2 ± 0.68	10.0 ± 1.30
Creatinine (mg/dL)	0.76 ± 0.052	0.78 ± 0.048
Bilirubin (mg/dL)	0.42 ± 0.041	0.44 ± 0.021
ALP (K‐A)	5.0 ± 0.58	5.5 ± 0.57
Albumin (g/dL)	4.1 ± 0.20	4.3 ± 0.29
TC (mg/dL)	136.7 ± 15.76	131.7 ± 9.16
HDL‐C (mg/dL)	48.3 ± 3.54	49.4 ± 3.44
TG (mg/dL)	130.1 ± 22.37	128.2 ± 12.83

*Note:* The table summarizes liver and kidney function markers, such as AST, ALT, BUN, and creatinine, which were not significantly different compared with the normal group, suggesting no hepatotoxicity or nephrotoxicity. Lipid profiles, including total cholesterol (TC), HDL‐C, and triglycerides (TG), also were not significantly different compared with the normal group, supporting the absence of metabolic disruptions. Data are presented as mean ± SD (*n* = 5). No significant differences were observed compared with the normal group (*p* > 0.05).

Abbreviations: ALP, Alkaline phosphatase; ALT, alanine aminotransferase; AST, aspartate aminotransferase; BUN, blood urea nitrogen; HDL‐C, high‐density lipoprotein cholesterol; TC, total cholesterol; TG, triglyceride.

## 4 Discussion

This investigation represents the first comprehensive evaluation of intravenous pharmacokinetics and acute safety for purified tryptanthrin, providing essential preclinical data that bridges traditional medicinal knowledge with modern pharmacological development. The pharmacokinetic profile demonstrates rapid distribution and biphasic elimination kinetics characteristic of lipophilic compounds with high tissue affinity. These findings are consistent with the physicochemical properties expected for indoloquinazoline alkaloids and support the traditional preference for *Indigo naturalis* in acute inflammatory conditions requiring rapid therapeutic intervention (Jeong et al. 2020; Parker and Davey 1992; Singh 2006).

The observed pharmacokinetic parameters reveal important characteristics relevant to the ethnopharmacological development of tryptanthrin. The large steady‐state volume of distribution (V_ss_ 23.1 L/kg) indicates extensive penetration into tissues. From a therapeutic perspective, this suggests that tryptanthrin may achieve effective concentrations in target tissues (e.g., skin or inflamed mucosa) even when plasma levels decline, which provides a pharmacokinetic rationale for its efficacy in tissue‐specific inflammatory diseases like psoriasis and colitis. Simultaneously, the rapid total body clearance (109 mL/min/kg, equivalent to 6.54 L/h/kg) suggests that the compound is efficiently eliminated from the systemic circulation. The large V_ss_ implies extensive tissue penetration, which is likely attributable to the high lipophilicity of tryptanthrin. However, the rapid systemic clearance observed in this study minimizes the duration of tissue exposure, potentially contributing to a favorable safety profile by reducing the risk of systemic accumulation.

This disposition profile is consistent with the rapid anti‐inflammatory effects historically reported for *Indigo naturalis*, where rapid onset of action has been empirically observed in clinical practice. The terminal half‐life of approximately 4 h suggests that multiple daily dosing may be required to maintain steady‐state plasma concentrations, consistent with traditional dosing regimens. However, the extensive tissue distribution may result in prolonged tissue residence times that extend pharmacological effects beyond plasma elimination (Jeong et al. 2020). While specific metabolic pathways were not evaluated in the present study, the rapid clearance rate is likely driven by efficient hepatic metabolism, primarily through cytochrome P450 enzymes such as CYP1A2, as reported previously (Jahng 2013; Sun et al. 2021). This metabolic pathway may also explain the individual variability historically observed in traditional medicine practice, potentially due to genetic polymorphisms in CYP1A2 activity affecting tryptanthrin clearance.

Critically, the acute safety evaluation revealed a favorable toxicity profile that markedly contrasts with the adverse events associated with crude *Indigo naturalis* preparations in clinical settings, such as PAH and liver injury (Hiraide et al. 2021; Masaki et al. 2021; Misumi et al. 2019; Naganuma et al. 2019; Nishio et al. 2016; Nishio et al. 2018). The absence of hepatotoxicity, as evidenced by normal AST, ALT, and bilirubin levels, strongly suggests that the hepatic dysfunction observed with crude preparations results from other chemical constituents (e.g., indigo, indirubin) rather than tryptanthrin itself. Although this study was limited to acute toxicity following a single dose, these findings support the ethnopharmacological strategy of isolating active principles to preserve therapeutic benefits while eliminating toxicity‐causing components. Similarly, the preservation of normal renal function parameters indicates that purified tryptanthrin does not contribute to nephrotoxic potential. Collectively, despite the substantial systemic exposure indicated by high C_max_ and AUC values, no significant alterations in these hepatic or renal biomarkers were observed, suggesting a wide safety margin at this therapeutic dose. Therefore, these safety findings validate the approach of compound isolation as a means of improving the therapeutic index of traditional medicine‐derived therapeutics.

The pharmacological activities previously documented for tryptanthrin, including potent antimicrobial (Duca et al. 2019; Yang et al. 2024), anti‐inflammatory (He et al. 2024; Pergola et al. 2012; Micallef et al. 2002; Kim et al. 2024), and anticancer effects (Liao et al. 2013; Zhou 2024; Chang et al. 2015), provide a strong scientific foundation supporting its modern clinical potential. The rapid systemic exposure achieved through intravenous administration positions tryptanthrin as particularly suitable for acute inflammatory conditions where traditional medicine practitioners have historically favored *Indigo naturalis* for its perceived rapid therapeutic onset.

There are limitations to this study that should be addressed in future research. First, the study employed a single‐dose design at 30 mg/kg. Although toxicological thresholds such as the maximum tolerated dose (MTD) or lethal dose (LD_50_) were not established in this study, the absence of acute toxicity at a therapeutically relevant dose (30 mg/kg) suggests a potentially wide safety margin compared with the effective dose range. While this approach successfully established the safety margin at an effective dose, future ethnopharmacological investigations should include dose‐escalation studies and chronic dosing regimens to provide a more comprehensive assessment of safety (Sun et al. 2021; Kirpotina et al. 2020). Second, the investigation was conducted in a single animal species, requiring further interspecies validation.

Future studies should also focus on pharmacokinetic‐pharmacodynamic (PK/PD) correlation. Correlating the observed plasma concentrations with specific biological effects is essential for defining a therapeutic window and establishing clinically relevant dosing regimens that bridge traditional empirical knowledge with modern evidence‐based medicine (Meibohm and Derendorf 1997). Such studies would validate whether the therapeutic concentrations historically achieved through traditional preparation methods are comparable with those observed in this purified model.

Taken together, these results highlight the potential of tryptanthrin as a promising candidate for further pharmaceutical development. The combination of rapid tissue distribution, efficient systemic elimination, and a favorable safety profile—devoid of the hepatotoxicity associated with the crude extract—supports its continued investigation in disease‐specific models. This study serves as a successful example of ethnopharmacology‐guided drug development, demonstrating how modern toxicological evaluation can validate and refine traditional medicinal knowledge for contemporary therapeutic applications.

## 5 Conclusions

This study provides the first systematic characterization of the intravenous pharmacokinetics and acute safety of purified tryptanthrin, establishing essential preclinical data that support its development as a safer therapeutic alternative to *Indigo naturalis*. Following intravenous administration, tryptanthrin exhibited rapid distribution into tissues (V_ss_ 23.1 L/kg) and efficient systemic elimination (CL 6.54 L/h/kg) with a terminal half‐life of approximately 4 h. Most importantly, the acute safety evaluation confirmed that tryptanthrin, at a therapeutically relevant dose, does not induce hepatotoxicity or nephrotoxicity frequently associated with crude *Indigo naturalis* preparations. These findings validate the ethnopharmacological rationale for isolating bioactive constituents to preserve therapeutic efficacy while eliminating toxicity‐causing components, providing a solid scientific foundation for future dose optimization and clinical translation.

## Author Contributions

**Hyung Jin Kim:** writing – original draft, investigation, visualization, formal analysis. **Gabsik Yang:** writing – review and editing, funding acquisition, project administration, resources, methodology. **Jun Ho Lee:** writing – review and editing, supervision, project administration, formal analysis, validation, conceptualization.

## Funding

This research was supported by the National Research Foundation (NRF), Republic of Korea (grant number: RS‐2023‐00262733, RS‐2025‐24535474).

## Conflicts of Interest

The authors declare no conflicts of interest.

## Data Availability

Data will be made available on request.
